# Bayesian Variable Selection and Estimation in Semiparametric Simplex Mixed-Effects Models with Longitudinal Proportional Data

**DOI:** 10.3390/e24101466

**Published:** 2022-10-14

**Authors:** Anmin Tang, Xingde Duan, Yuanying Zhao

**Affiliations:** 1 Yunnan Key Laboratory of Statistical Modeling and Data Analysis, Yunnan University, Kunming 650091, China; 2 Department of Mathematics and Statistics, Guizhou University of Finance and Economics, Guiyang 550025, China; 3 College of Mathematics and Information Science, Guiyang University, Guiyang 550005, China

**Keywords:** simplex distribution, Gibbs sampler, Metropolis–Hastings algorithm, Dirichlet process prior, Bayesian Lasso

## Abstract

In the development of simplex mixed-effects models, random effects in these mixed-effects models are generally distributed in normal distribution. The normality assumption may be violated in an analysis of skewed and multimodal longitudinal data. In this paper, we adopt the centered Dirichlet process mixture model (CDPMM) to specify the random effects in the simplex mixed-effects models. Combining the block Gibbs sampler and the Metropolis–Hastings algorithm, we extend a Bayesian Lasso (BLasso) to simultaneously estimate unknown parameters of interest and select important covariates with nonzero effects in semiparametric simplex mixed-effects models. Several simulation studies and a real example are employed to illustrate the proposed methodologies.

## 1. Introduction

Various mixed-effects models based on simplex distribution have increasingly become popular tools in the analysis of longitudinal continuous proportional data over time in many biological, medical and clinical studies. Under the framework of generalized linear mixed models, see Qiu et al. [1] for information on developing a simplex generalized linear mixed model on the basis of the penalized quasi-likelihood (PQL) and restricted maximum likelihood (REML) inference; see Zhang and Wei [2] for information on using the maximum likelihood estimation combining the stochastic approximation (SA) algorithm and the MCMC method to infer on simplex distribution nonlinear mixed models; see Zhao et al. [3] for information on implementing the MCMC algorithm to obtain the joint Bayesian estimate of simplex distribution nonlinear mixed models from the Bayesian perspective; see Bonat et al. [4] for information on investigating the likelihood analysis for a class of simplex mixed models with logit, probit, complement log–log and Cauchy link functions; see Quintero [5] for information on presenting the sensitivity analysis for variance parameters of random effects in Bayesian simplex mixed models. The random effects in the abovementioned mixed-effects models are assumed to have a multivariate normal distribution. However, in some practical applications, it is questionable for the normal assumption for random effects to analyze the skewed, bimodal and heavy-tailed longitudinal data. Therefore, it is essential to incorporate a semiparametric hierarchical structure via a Dirichlet process prior distribution for the random effects into the simplex mixed-effects models to accommodate longitudinal proportional data.

The nonparametric Bayesian approach based on Dirichlet process (DP) prior for random effects in mixed-effects models has been receiving a lot of attention in recent years. For example, Kleinman and Ibrahim [6] used a Dirichlet process prior for the general distribution of the random effects in generalized linear mixed model. As a variant of Dirichlet process prior, the truncation approximation Dirichlet process with stick-breaking priors is widely incorporated into various mixed-effects models to specify the general distribution of random effects. For example, Tang and Duan [7] used this approach for a semiparametric Bayesian approach to generalized partial linear mixed model; Tang and zhao [8] used this approach for nonlinear reproductive dispersion mixed models; Zhao et al. [9] used this approach for a semiparametric Bayesian approach to binomial distribution logistic mixed-effects model. In particular, Duan et al. [10] used a truncated and centered Dirichlet process prior to specify random effects in semiparametric reproductive dispersion mixed model. However, the abovementioned DP with stick-breaking prior for random effects is inappropriate when the underlying density of random effects is continuous. In addition, this type of variant for Dirichlet process prior is rather time-consuming in the calculation process for complicated models. Therefore, to address the above issues, the goal of this paper is to propose a new semiparametric simplex mixed-effects models with the random effects distribution specified by the centered Dirichlet process mixture model (CDPMM).

Although various methodologies have been developed to make statistical inference on the aforementioned simplex mixed-effects models, little work has been performed for the variable selection of simplex mixed-effects models. Classical model-selection methods, such as the step-wise selection method [11], the model comparison via Bayes factor [12], the Akaike information criterion [13] and Deviance information criterion [14], are often used to identify the important covariates in regression analysis; however, these approaches are generally computationally intensive and unstable for complicated mixed models with many covariates. On the other hand, the regularization (penalization) method has increasingly become a popular tool for conducting variable selection in regression analysis. Commonly used regularization methods in the context of linear regression include least absolute shrinkage and selection operator (Lasso) [15], elastic net [16] and adaptive lasso [17]. In addition, Park and Casella [18] proposed the Bayesian version of the Lasso (BLasso) by assigning the conditional Laplace prior of regression coefficients and the gamma distribution of shrinkage parameter under the Bayesian framework. The BLasso procedure has been extended to various complex models including semiparametric structural equation models [19] and semiparametric joint models of multivariate longitudinal and survival data [20]. In particular, Erd et al. [21] pointed out that Bayesian penalization methods perform similarly or sometimes even better than frequentist penalization methods, since Bayesian penalization methods can easily provide credible intervals (CIs) for parameters of interest and obtain the estimate of the penalty parameter by assigning an appropriate prior distribution. Therefore, the other main purpose of this paper is to extend the BLasso procedure to the considered semiparametric simplex mixed-effects models.

The paper is organized as follows: In Section 2, we propose a new semiparametric simplex mixed-effects models with random effects following the centered Dirichlet process mixture model (CDPMM) and incorporate a BLasso procedure into the proposed simplex mixed-effects models. The required conditional distributions are derived in Section 3. Two simulation studies and a real example are used to illustrate the proposed methodologies in Section 4. Some concluding remarks are given in Section 5.

## 2. Model and Notation

The simplex distribution was firstly proposed by Barndorff-Nielsen and Jørgensen [22], whose probability density function is specified as

(1) $$p\left(y;\mu ,\sigma^{2}\right)=\left\{\begin{array}{cc} {\left[2\pi \sigma^{2}{\left\{y\left(1-y\right)\right\}}^{3}\right]}^{-1/2}exp\left\{-\frac{d(y;\mu )}{2\sigma^{2}}\right\}, & if\;0<y<1\\ 0, & otherwise \end{array}\right.$$

where μ∈(0,1) denotes the mean parameter; $\sigma^{2}>0$ represents the dispersion parameter; and $d\left(y;\mu \right)=\frac{{\left(y-\mu \right)}^{2}}{y\left(1-y\right)\mu^{2}{\left(1-\mu \right)}^{2}}$. For simplicity of notation, we denote $y∼S^{-}\left(\mu ,\sigma^{2}\right)$ if a random variable, *y*, is distributed as a simplex distribution with mean parameter, μ, and dispersion parameter, $\sigma^{2}$, in the rest of this paper.

In the context of longitudinal data analysis, let $y_{ij}$ denote the longitudinal percentage outcome for the *i*th individual at the *j*th follow-up time $t_{ij}$, and $0<y_{ij}<1$, $i=1,\ldots ,n,j=1,\ldots ,n_{i}$. We assume that, given a q×1 random effects $b_{i}$ corresponding to the *i*th individual, the responses $y_{ij}$ are conditionally independent and each $y_{ij}|b_{i}$ is distributed as a simplex distribution with conditional means, $\mu_{ij}=E(y_{ij}\left|b_{i}\right)$, and constant dispersion parameter, $\sigma^{2}$: that is, $y_{ij}|b_{i}∼S^{-}\left(\mu_{ij},\sigma^{2}\right)$. Under the framework of GLMM, the conditional mean is linked to explanatory variables and random effects as follows:

(2) $$f\left(\mu_{ij}\right)\overset{\Delta }{=}\eta_{ij}=x_{ij}^{T}\beta +z_{ij}^{T}b_{i},$$

where an unknown and monotone link function f(·) is chosen as the logit link; $x_{ij}$ is a (p+1)×1 vector of covariates which consist of the constant 1 and time-dependent covariates observed at time point $t_{ij}$; β is a (p+1)×1 vector of unknown regression parameters; $z_{ij}$ is a q×1 vector of time-dependent variables which may include some elements of $x_{ij}$ corresponding to random effects $b_{i}$. In classical random-effects models, the random effects in (2) are generally assumed to be a multivariate normal distribution, which may give rise to biased estimates of parameters or even misleading conclusions. Thus, inspired by Ohlssen and Spiegelhalter [23], we used the DP mixture of normals to specify the random effects: that is, $b_{i}\overset{i.i.d.}{∼}\sum_{g=1}^{\infty }\pi_{g}N_{q}\left(\mu_{g},\Omega_{g}\right)$ with $(\mu_{g},\Omega_{g})∼P$, where P is an unknown random probability. Clearly, it is rather difficult and inefficient to make Bayesian estimates for regression parameter β and dispersion parameter $\sigma^{2}$ in Equation (2) since an unknown form of P is involved. To address the difficulty, the Dirichlet process (DP) prior is usually introduced to approximate P, i.e., $P∼\mathrm{DP}(\tau F_{0})$, in which $F_{0}$ is a given base distribution such as multivariate normal distribution that serves as a starting point for constructing the nonparametric distribution, and τ is a weight that indicates the researcher’s certainty of $F_{0}$ as the distribution of P. In particular, Sethuraman [24] showed that the DP prior $\mathrm{DP}(\tau F_{0})$ has the stick-breaking prior representation; however, this approach causes a nonzero mean of random effects [25] and a discrete probability distribution of random effects [23]. Generally, the variants of Dirichlet Process proposed by Ishwaran and Zarepour [26] and Yang et al. [25] were regarded as discrete Dirichlet processes (discrete DPs). A discrete DP with stick-breaking prior for random effects is inappropriate when the underlying density of random effects is continuous. Furthermore, violation of zero mean assumption on the random effects may lead to non-identifiability in the aforementioned random effects model. In addition, the discrete DP methods with stick-breaking prior for random effects are generally computationally intensive for the complicated models.

To overcome the above issues, inspired by Ohlssen and Spiegelhalter [23] and Yang et al. [25], we incorporated the following variant of Dirichlet process into the above model in (2) to specify random effects. That is,

(3) $$b_{i}\overset{i.i.d.}{∼}\sum_{g=1}^{\infty }\pi_{g}N_{q}\left(\mu_{g},\Omega_{g}\right)\;\;\mathrm{with}\;\;\mu_{g}=\mu_{g}^{*}-\sum_{g=1}^{\infty }\pi_{g}\mu_{g}^{*}\;\;\mathrm{and}\;\;\left(\mu_{g}^{*},\Omega_{g}\right)\overset{i.i.d.}{∼}F_{0},$$

where $\pi_{g}$ is a random probability weight satisfying $0\leq \pi_{g}\leq 1$ and $\sum_{g=1}^{\infty }\pi_{g}=1$. In addition, $\pi_{g}$ is assumed to be be independent of $\left(\mu_{g}^{*},\Omega_{g}\right)$. This variant of Dirichlet process is referred to as the centered Dirichlet process mixture model (CDPMM). As in Ishwaran and Zarepour [26], we adopt the following mixture model of the truncated approximation DP for P:

(4) $$b_{i}\overset{i.i.d.}{∼}\sum_{g=1}^{G}\pi_{g}N_{q}\left(\mu_{g},\Omega_{g}\right)\;\;\mathrm{with}\;\;\mu_{g}=\mu_{g}^{*}-\sum_{g=1}^{G}\pi_{g}\mu_{g}^{*}\;\;\mathrm{and}\;\;\left(\mu_{g}^{*},\Omega_{g}\right)\overset{i.i.d.}{∼}F_{0},$$

where *G* is a limited integer satisfying 1≤G<∞. As for the selection of *G*, Ishwaran and Zarepour [26] pointed out that a moderate value of *G* such as 25 may be enough to capture a good approximation in practical application. Thus, the value of *G* is chosen to be 25 in the rest of this paper. Furthermore, the random probability weight, $\pi_{g}$, is specified by the following stick-breaking procedure:

(5) $$\pi_{1}=\vartheta_{1}\;\;\mathrm{and}\;\;\pi_{g}=\vartheta_{g}\prod_{\iota =1}^{g-1}\left(1-\vartheta_{\iota }\right)\;\;\mathrm{for}\;g=2,\ldots ,G,$$

where $\vartheta_{g}\overset{i.i.d.}{∼}\mathrm{Beta}\left(1,\tau \right)$ for g=1,…,G−1, and $\vartheta_{G}=1$ so that $\sum_{g=1}^{G}\pi_{g}=1$. The prior distribution for the unknown parameter τ is chosen as $\tau ∼\Gamma (a_{1},a_{2})$, such that the posterior distribution for τ is conjugated. Here, we set the hyperparameters $a_{1}$ and $a_{2}$ to be 25 and 5, respectively, such that large value of τ is generated, which results in more unique $b_{i}$ values.

It is rather difficult and inefficient to generate observations from posterior distributions of $b_{i}$ with the above DP prior via MCMC algorithm. Furthermore, a latent variable $L_{i}\in \left\{1,\ldots ,G\right\}$ is introduced to solve sample issue since this latent variable can record each $b_{i}$’s cluster membership and convey its parametric value to the distribution of $b_{i}$. Let $L=\{L_{1},\ldots ,L_{n}\}$, $\pi =\{\pi_{1},\ldots ,\pi_{G}\}$, $\mu^{*}=\left\{\mu_{1}^{*},\ldots ,\mu_{G}^{*}\right\}$ and $\Omega =\{\Omega_{1},\ldots ,\Omega_{G}\}$, in which $\Omega_{g}=\mathrm{diag}\left(\omega_{g1},\ldots ,\omega_{gq}\right)$ for g=1,…,G. As in Ishwaran and Zarepour [26], the hierarchical structure defined in (4) can be written as

(6) $$L_{i}|\pi \overset{i.i.d}{∼}\sum_{g=1}^{G}\pi_{g}\delta_{g}\left(\cdot \right)\;\;\mathrm{and}\;\;\left(\pi ,\mu^{*},\Omega \right)∼f_{1}\left(\pi \right)f_{2}\left(\mu^{*}\right)f_{3}\left(\Omega \right),$$

where $\delta_{g}\left(\cdot \right)$ denotes a discrete probability measure concentrated at *g*, $f_{1}\left(\pi \right)$ is defined in Equation (5), the prior for $\mu_{g}^{*}$ associated with $f_{2}\left(\mu^{*}\right)=\prod_{g=1}^{G}f_{2}\left(\mu_{g}^{*}\right)$ is defined by

(7) $$\mu_{g}^{*}|\xi ,\Psi \overset{i.i.d}{∼}N_{q}\left(\xi ,\Psi \right),\;\xi \left|\xi^{0},\Psi^{0}∼N_{q}\left(\xi^{0},\Psi^{0}\right),\;\psi_{j}^{-1}\right|c_{1},c_{2}∼\Gamma \left(c_{1},c_{2}\right)\;\;\mathrm{for}\;\;j=1,\ldots ,q,$$

and the prior for $\omega_{g_{j}}$ related to $f_{3}\left(\Omega \right)=\prod_{g=1}^{G}\prod_{j=1}^{q}f_{3}\left(\omega_{g_{j}}\right)$ is defined by

(8) $$\omega_{g_{j}}^{-1}\left|\omega_{j}^{a},\varpi_{j}∼\Gamma \left(\omega_{j}^{a},\varpi_{j}\right)\;\;\mathrm{and}\;\;\varpi_{j}\right|\varpi_{j}^{a},\varpi_{j}^{b}∼\Gamma \left(\varpi_{j}^{a},\varpi_{j}^{b}\right),$$

where $\Psi =\mathrm{diag}(\psi_{1},\ldots ,\psi_{q})$, $\Gamma (c_{1},c_{2})$ denotes the Gamma distribution with parameters $c_{1}$ and $c_{2}$, and $\xi^{0},\Psi^{0},c_{1},c_{2},\omega_{j}^{a},\varpi_{j}^{a}$ and $\varpi_{j}^{b}$ are pre-specified hyperparameters: that is, $\xi^{0}=0_{q\times 1}$, $\Psi^{0}=I_{q}$, $c_{1}=11$, $c_{2}=2.5$, $\omega_{j}^{a}=3$, $\varpi_{j}^{a}=n$ and $\varpi_{j}^{b}=10$. Thus, given the values of $L_{i}$, $\mu^{*}$ and Ω, the prior for random effect $b_{i}$ is assumed to be $N_{q}\left(\mu_{L_{i}},\Omega_{L_{i}}\right)$ with $\mu_{L_{i}}=\mu_{L_{i}}^{*}-\Sigma_{g=1}^{G}\mu_{g}^{*}$.

To estimate the unknown parameters β and $\sigma^{2}$ in Equation (2) from the Bayesian perspective, it is necessary to specify priors for β and $\sigma^{2}$. In order to alleviate the computational burden, the conjugate prior distribution for dispersion parameter $\sigma^{2}$ is taken to be

(9) $$\sigma^{-2}∼\Gamma \left(\sigma_{a}^{2},\sigma_{b}^{2}\right),$$

where the values of hyperparameters $\sigma_{a}^{2}$ and $\sigma_{b}^{2}$ are taken to be 1 and 0.01, respectively. In this paper, the main goal is to incorporate the Bayesian version of lasso into our proposed model (2) to conduct parameter estimation and model selection simultaneously. Similar to Park and Casella [18] and Tang et al. [20], the following Laplace prior on β is given by

$$\pi \left(\beta \right)=\prod_{k=0}^{p}\frac{\nu }{2}\exp\left(-\nu |\beta_{k}|\right),$$

where ν is the regularization parameter. Because the mass of the above presented Laplace prior is quite highly concentrated around zero with a distinct peak at zero, posterior means or modes of $\beta_{k}$’s are shrunk towards zero, which is the key principle in using BLasso method to select the important covariates. Following Robert [15], the Laplace distribution with the form $a\exp\left(-a|x|\right)/2$ can be represented as a scale mixture of normal distributions with independent exponentially distributed variance: that is,

$$\frac{a}{2}\exp\left(-a|x|\right)=\int_{0}^{\infty }\frac{1}{\sqrt{2\pi u}}\exp\left(-\frac{x^{2}}{2u}\right)\frac{a^{2}}{2}\exp\left(-\frac{a^{2}u}{2}\right)du,\;\;\mathrm{for}\;a>0.$$

Therefore, the aforementioned prior for β can be reformulated as the following hierarchical structure:

(10) $$\begin{array}{ccc} \; & \beta |H_{\beta }∼N_{p}\left(0,H_{\beta }\right)\;with\;H_{\beta }=\mathrm{diag}\left(h_{\beta_{0}}^{2},\ldots ,h_{\beta_{p}}^{2}\right), & \\ \; & h_{\beta_{0}}^{2},\ldots ,h_{\beta_{p}}^{2}∼\prod_{j=0}^{p}\frac{\nu^{2}}{2}\exp\left(-\frac{\nu^{2}}{2}h_{\beta_{k}}^{2}\right)\\ \; & \nu^{2}∼\mathrm{Gamma}\left(\nu_{a}^{2},\nu_{b}^{2}\right), & \end{array}$$

where the hyperparameters $\nu_{a}^{2}$ and $\nu_{b}^{2}$ are selected as 1 and 0.1, respectively, which imply diffuse prior. Similar to Park and Casella [18], the posterior distribution for $h_{\beta_{k}}^{2}$ and $\nu^{2}$ in the hierarchical structure (10) have closed expressions, such that this hierarchical representation greatly simplifies the computation. Therefore, it follows from Equation (10) that the posterior distribution of $\nu^{2}$ is distributed as the following Gamma distribution

(11) $$\nu^{2}|\beta_{k},H_{\beta_{k}}∼\mathrm{Gamma}\left(\nu_{a}^{2}+p+1,\nu_{b}^{2}+\frac{1}{2}\sum_{k=0}^{p}h_{\beta_{k}}^{2}\right).$$

In addition, the posterior distributions for $h_{\beta_{0}}^{2},\ldots ,h_{\beta_{p}}^{2}$ are derived as

(12) $$h_{\beta_{k}}^{-2}|\beta_{k},\nu^{2}∼\mathrm{IG}\left(\left|\frac{\nu }{\beta_{k}}\right|,\nu^{2}\right)\;\;\mathrm{for}\;\;k=0,\ldots ,p,$$

where IG(a,b) denotes the inverse Gaussian distribution with parameter *a* and the shape parameter *b*. As for sampling from the inverse Gaussian distribution, Tang et al. [20] gave a detailed procedure.

## 3. Bayesian Analysis of Model

Let $Y=\{y_{ij}:i=1,\ldots ,n,j=1,\ldots ,n_{i}\}$, $X=\{x_{ij}:i=1,\ldots ,n,j=1,\ldots ,n_{i}\}$, $Z=\{z_{ij}:i=1,\ldots ,n,j=1,\ldots ,n_{i}\}$, and random effects $b=\{b_{i}:i=1,\ldots ,n\}$. To obtain joint Bayesian estimates of unknown parameters β and $\sigma^{2}$ and the random effects, as well as to select important covariates in our considered models, a hybrid algorithm combining the block Gibbs sampler and the Metropolis–Hastings algorithm is employed to draw a sequence of random observations from the joint posterior distribution $p(\beta ,\sigma^{2},b|Y,X,Z)$, as follows. In this hybrid algorithm, observations $\left\{\beta ,\sigma^{2},b\right\}$ are iteratively drawn from the following conditional distributions: $p(\beta |\sigma^{2},b,Y,X,Z)$, $p(\sigma^{-2}|\beta ,b,Y,X,Z)$ and $p(b|\beta ,\sigma^{2},Y,X,Z)$.

**Block Gibbs Sampler (A): Conditional distribution related to** β

It follows from Equations (2) and (10) that the conditional distribution $p(\beta |\sigma^{2},b,Y,X,Z)$ is proportional to

$$\exp\left\{-\frac{1}{2}\left[\sum_{i=1}^{n}\sum_{j=1}^{n_{i}}\frac{1}{\sigma_{i}^{2}}d\left(y_{ij};\mu_{ij}\right)+{\left(\beta -\beta_{0}\right)}^{T}H_{\beta }^{-1}\left(\beta -\beta_{0}\right)\right]\right\},$$

which is an unfamiliar distribution. Therefore, we used the well-known Metropolis–Hastings (MH) algorithm to generate observations from the aforementioned conditional distribution as follows. Given the current value $\beta^{\left(l\right)}$, new candidate β is generated from the proposal distribution $N(\beta^{\left(l\right)},\sigma_{\beta }^{2}\Sigma_{\beta })$ and is accepted with probability

$$min\left\{1,\frac{p(\beta |\sigma^{2},b,Y,X,Z)}{p(\beta^{\left(l\right)}|\sigma^{2},b,Y,X,Z)}\right\},$$

where

$$\Sigma_{\beta }={\left(\sum_{i=1}^{n}\sum_{j=1}^{n_{i}}\frac{\ddot{d}\left(y_{ij};{\bar{\mu }}_{ij}\right)}{2\sigma^{2}{\left\{\dot{f}\left(\mu_{ij}\right)\right\}}^{2}}x_{ij}^{T}x_{ij}+H_{\beta }^{-1}\right)}^{-1}$$

with $\dot{f}\left(\mu_{ij}\right)=\partial f/\partial \mu_{ij}$ and $\ddot{d}\left(y_{ij};{\bar{\mu }}_{ij}\right)=E_{y_{ij}}\left(\partial^{2}d\left(y_{ij};\mu_{ij}\right)/\partial \mu_{ij}^{2}\right)\left|{}_{\beta =\beta^{\left(l\right)}}\right.$, and the variance coefficient $\sigma_{\beta }^{2}$ can be chosen, such that the average acceptance rates are approximately 0.25 or more.

**Block Gibbs Sampler (B): Conditional distribution related to** $\sigma^{-2}$

The conditional distribution $p(\sigma^{-2}|\beta ,b,Y,X,Z)$ can be derived as

$$p\left(\sigma^{-2}|\beta ,b,Y,X,Z\right)\propto {\left(\sigma^{-2}\right)}^{0.5\sum_{i=1}^{n}n_{i}+\sigma_{a}^{2}-1}\exp\left\{-\left(0.5\sum_{i=1}^{n}\sum_{j=1}^{n_{i}}d\left(y_{ij};\mu_{ij}\right)+\sigma_{b}^{2}\right)\sigma^{-2}\right\},$$

which can be simplified as

$$\sigma^{-2}|\beta ,b,Y,X,Z∼\Gamma \left(0.5\sum_{i=1}^{n}n_{i}+\sigma_{a}^{2},0.5\sum_{i=1}^{n}\sum_{j=1}^{n_{i}}d\left(y_{ij};\mu_{ij}\right)+\sigma_{b}^{2}\right).$$

Clearly, it is straightforward and efficient to draw observations for $\sigma^{-2}$ from the Gamma distribution via any statistical software.

**Block Gibbs Sampler (C): Conditional distribution related to $\theta_{b}$**

Let $\theta_{b}$ denote all unknown parameters associated with distribution of random effects $b_{i}$, i=1,…,n. $\theta_{b}$ can be iteratively sampled by using the following nine steps:

*Step* (a). Conditional distribution of ξ given $\left(\mu^{*},\Psi ,b\right)$ is given

$$\xi |\mu^{*},\Psi ,b∼N_{q}\left(A,B\right)$$

where $B={\left(G\Psi^{-1}+{\left(\Psi^{0}\right)}^{-1}\right)}^{-1}$ and $A=B({\left(\Psi^{0}\right)}^{-1}\xi^{0}+\Psi^{-1}\sum_{g=1}^{G}\mu_{g}^{*})$.

*Step* (b). For j=1,…,q, the diagonal elements of Ψ is conditionally distributed as

$$\psi_{j}^{-1}|\mu^{*},\xi ∼\Gamma \left(c_{1}+\frac{G}{2},c_{2}+\frac{1}{2}\sum_{g=1}^{G}{\left(\mu_{g_{j}}^{*}-\xi_{j}\right)}^{2}\right),$$

where $\mu_{g_{j}}^{*}$ is the *j*th element of $\mu_{g}^{*}$ and $\xi_{j}$ is the *j*th element of ξ.

*Step* (c). For j=1,…,q, $\varpi_{j}|\Omega$ is conditionally distributed as

$$\varpi_{j}|\Omega ∼\Gamma \left(\varpi_{j}^{a},\varpi_{j}^{b}+\sum_{g=1}^{G}\omega_{g_{j}}^{-1}\right),$$

where $\omega_{g_{j}}$ is the jth diagonal element of $\Omega_{g}$.

*Step* (d). Following Ishwaran and Zarepour [26], the conditional distribution of τ|π can be expressed as

$$\tau |\pi ∼\Gamma \left(a_{1}+G-1,a_{2}-\sum_{g=1}^{G-1}\log\left(1-\nu_{g}^{*}\right)\right),$$

where $\nu_{g}^{*}$ is a random weight sampled from the beta distribution and it is sampled with step (e).

*Step* (e). It is easily obtained that the conditional distribution of π|L,τ is distributed as the following generalized Dirichlet distribution:

$$\pi |L,\tau ∼\mathrm{Dir}(a_{1}^{*},b_{1}^{*},\ldots ,a_{G-1}^{*},b_{G-1}^{*}),$$

where $a_{g}^{*}=1+d_{g},b_{g}^{*}=\tau +\sum_{\iota =g+1}^{G}d_{\iota }$ for g=1,…,G−1, and $d_{g}$ is the number of $L_{i}^{\prime }s$(and thus individuals) whose values equal to *g*. Simulating observation from the conditional distribution π|L,τ can be conducted as follows. First, $\nu_{g}^{*}$ is independently generated from a Beta distribution $\left(a_{g}^{*},b_{g}^{*}\right)$. Then, $\pi_{1},\ldots ,\pi_{G}$ are obtained from the following formulae:

$$\pi_{1}=\nu_{1}^{*},\;\;\pi_{G}=1-\sum_{g=1}^{G-1}\pi_{g},\;\;\mathrm{and}\;\;\pi_{g}=\prod_{\iota =1}^{g-1}\left(1-\nu_{\iota }^{*}\right)\nu_{g}^{*},\;\;\mathrm{for}\;g\neq 1\;\;\mathrm{or}\;\;G.$$

*Step* (f). Conditional distribution of $\mu^{*}|\xi ,\Psi ,\Omega ,L,b$.

Let $L_{1}^{*},\ldots ,L_{d}^{*}$ be the *d* unique values of $\left\{L_{1},\ldots ,L_{n}\right\}$ (i.e., unique number of “clusters”), for g=1,…,G; $\mu_{g}^{*}$ is conditionally distributed as follows:

$$\mu_{g}^{*}|\xi ,\Psi ∼N_{q}\left(\xi ,\Psi \right)\;\;\mathrm{for}\;\;g\notin \left\{L_{1}^{*},\ldots ,L_{d}^{*}\right\},$$

$$\mu_{g}^{*}|\xi ,\Psi ,\Omega ,L,b∼N_{q}\left(E_{g},F_{g}\right)\;\;\mathrm{for}\;\;g\in \left\{L_{1}^{*},\ldots ,L_{d}^{*}\right\},$$

where $F_{g}={\left(\Psi^{-1}+\Sigma_{\left\{i:L_{i}=g\right\}}\Omega_{i}^{-1}\right)}^{-1}$ and $E_{g}=F_{g}\left(\Psi^{-1}\xi +\Sigma_{\left\{i:L_{i}=g\right\}}\Omega_{i}^{-1}b_{i}\right)$ for $g\in \{L_{1}^{*},\ldots ,L_{d}^{*}\}$. Given $\mu_{g}^{*}$, $\mu_{g}=\mu_{g}^{*}-\Sigma_{g=1}^{G}\pi_{g}\mu_{g}^{*}$, $\mu^{*}=\left\{\mu_{1}^{*},\cdots ,\mu_{G}^{*}\right\}$ and $\mu =\{\mu_{1},\cdots ,\mu_{G}\}$.

*Step* (g). Conditional distribution of Ω|μ,ϖ,L,τ.

Similar to the notation of step (f), given *g*, for j=1,…,q, the jth diagonal element of $\Omega_{g}$ is conditionally distributed as

$$\omega_{g_{j}}∼\Gamma \left(\omega_{j}^{a},\varpi_{j}\right)\;\;\mathrm{for}\;\;g\notin \left\{L_{1}^{*},\ldots ,L_{d}^{*}\right\},$$

$$\omega_{g_{j}}∼\Gamma \left(\frac{d_{g}}{2}+\omega_{j}^{a},\varpi_{j}+\sum_{\left\{i:L_{i}=g\right\}}\frac{1}{2}{\left(b_{i_{j}}-\mu_{g_{j}}\right)}^{2}\right)\;\;\mathrm{for}\;\;g\in \left\{L_{1}^{*},\ldots ,L_{d}^{*}\right\},$$

where $b_{i_{j}}$ is the *j*th element of $b_{i}$ and $\mu_{g_{j}}$ is the *j*th element of $\mu_{g}$. Given $\omega_{g_{j}}$, $\Omega_{g}=\mathrm{diag}\left(\omega_{g_{1}},\ldots ,\omega_{g_{q}}\right)$ and $\Omega =\{\Omega_{1},\ldots ,\Omega_{G}\}$.

*Step* (h). The conditional distribution of $L_{i}|\pi ,\mu ,\Omega ,b$ is given by

$$L_{i}|\pi ,\mu ,\Omega ,b\overset{i.i.d}{∼}\mathrm{multinomial}\left(\pi_{ig}^{*},g=1,\ldots ,G\right),$$

where $\pi_{ig}^{*}$ is proportional to $\left(\pi_{g}p\left(b_{i}|\mu_{g},\Omega_{g}\right)\right)$ with $b_{i}|\mu_{g},\Omega_{g}∼N_{q}\left(\mu_{g},\Omega_{g}\right)$, and $\pi_{g}\left(g=1,\ldots ,G\right)$ are sampled from step (e). Given $L_{i}$, μ and Ω, the prior of $b_{i}$ is distributed as $N_{q}\left(\mu_{L_{i}},\Omega_{L_{i}}\right)$, with $\mu_{L_{i}}$ and $\Omega_{L_{i}}$ being the $L_{i}$ elements of sets μ and Ω, respectively.

*Step* (i). The conditional distribution for $b=\{b_{i}:i=1,\ldots ,n\}$

The conditional distribution $p(b_{i}|\beta ,\sigma^{2},Y,X,Z)$ is non-standard and cannot be derived directly via Gibbs sampling for i=1,⋯,n. Specifically,

$$p\left(b_{i}|\beta ,\sigma^{2},Y,X,Z\right)\propto p\left(b_{i}|\mu_{L_{i}},\Omega_{L_{i}}\right)p\left(Y_{i}|\beta ,\sigma^{2},b_{i},X,Z\right).$$

where $Y_{i}=\left\{y_{ij}:j=1,\ldots ,n_{i}\right\}$, $p\left(Y_{i}|\beta ,\sigma^{2},b_{i},X,Z\right)=\prod_{j=1}^{n_{i}}p\left(y_{ij};\mu ,\sigma^{2}\right)$ with $p(y_{ij};\mu ,\sigma^{2})$ specified by Equation (1) and μ by Equation (2). The Metropolis–Hastings algorithm used to sample observation $b_{i}$ is implemented as follows. At the *ℓ*th iteration with a current value $b_{i}^{\left(\ell \right)}$, a new candidate $b_{i}$ is drawn from the normal distribution $N_{q}\left(b_{i}^{\left(\ell \right)},\sigma_{b}^{2}\Sigma_{b_{i}}\right)$, where $\Sigma_{b_{i}}={\left(\Omega_{L_{i}}^{-1}+\Xi_{i}\right)}^{-1}$ and $\Xi_{i}=-\partial^{2}{\left(\ln\left(p\left(Y_{i}|\beta ,\sigma^{2},b_{i},X,Z\right)\right)/\partial b_{i}\partial b_{i}^{T}\right|}_{b_{i}=b_{i}^{\left(\ell \right)}}$. The new $b_{i}$ is accepted with probability

$$\min\left\{1,\frac{p\left(b_{i}|\mu_{L_{i}},\Omega_{L_{i}}\right)p\left(Y_{i}|\beta ,\sigma^{2},b_{i},X,Z\right)}{p\left(b_{i}^{\left(\ell \right)}|\mu_{L_{i}},\Omega_{L_{i}}\right)p\left(Y_{i}|\beta ,\sigma^{2},b_{i}^{\left(\ell \right)},X,Z\right)}\right\}.$$

The variance, $\sigma_{b}^{2}$, can be chosen such that the average acceptance rate is approximately 0.25 or more.

Then, we can obtain a series of sample observations—$\left\{\left(\beta^{\left(l\right)},{\sigma^{2}}^{\left(l\right)},b^{\left(l\right)}\right):l=1,2,\ldots ,L\right\}$—via the above iterative process. Then, Bayesian estimates of $\beta ,\sigma^{2}$ and $b_{i}$ for given *i* can be obtained by sample mean as follows:

$$\hat{\beta }=\frac{1}{L}\sum_{\ell =1}^{L}\beta^{\left(\ell \right)},\;\;\hat{\sigma^{2}}=\frac{1}{L}\sum_{\ell =1}^{L}{\sigma^{2}}^{\left(\ell \right)},\;\;{\hat{b}}_{i}=\frac{1}{L}\sum_{\ell =1}^{L}b_{i}^{\left(\ell \right)},$$

Similarly, the consistent estimates of the posterior covariance matrices of β and $\sigma^{2}$ can be obtained via the sample covariance matrices.

## 4. Numerical Examples

To investigate the behavior of our proposed model and the BLasso method under the Bayesian framework, we conduct four simulation studies and a real example related to a prospective ophthalmology study.

### 4.1. Simulation Study

In the first simulation study, we assume that, given the random effects $b_{i}={\left(b_{i1},b_{i2}\right)}^{T}$, the longitudinal percentage responses, $y_{ij}$, are conditionally independent and each $y_{ij}|b_{i}$ (i=1,…,100, j=1,…,6) follows the simplex distribution—that is, $y_{ij}|b_{i}∼S^{-}\left(\mu_{ij},\sigma^{2}\right)$. The conditional mean parameter $\mu_{ij}=E(y_{ij}\left|b_{i}\right)$ is specified as follows:

$$logit\left(\mu_{ij}\right)=x_{ij}^{T}\beta +z_{ij}^{T}b_{i}=\beta_{0}+x_{1ij}\beta_{1}+x_{2ij}\beta_{2}+x_{3ij}\beta_{3}+t_{ij}\beta_{4}+b_{i1}+t_{ij}b_{i2},$$

where $x_{1ij}$ randomly takes 1 or -1 with equal probability—$x_{2ij}\;\;\mathrm{and}\;\;x_{3ij}\overset{i.i.d}{∼}N\left(0,1\right)$, $t_{ij}=0.2j$ for j=0,…,5. Moreover, the true values of the parameters are specified as follows: $\beta ={\left(\beta_{0},\ldots ,\beta_{4}\right)}^{T}={\left(-0.45,0.00,0.45,0.00,0.45\right)}^{T}$. This implies that a covariate corresponding to 0 is unimportant, and that $\sigma^{2}=1$. The true distribution of random effect, $b_{i}$, is assumed to be

$$b_{i1}\overset{i.i.d}{∼}N\left(0,0.8\right),b_{i2}=b_{i2}^{*}-2\;\;\mathrm{with}\;\;b_{i2}^{*}\overset{i.i.d}{∼}\Gamma \left(4,2\right),$$

where the random effects cover the symmetric and skewed features with mean 0. A total of 500 Monte Carlo replications were conducted on the basis of the above-simulated setup.

In the second simulation study, 500 simulated datasets were generated by using the same setup as specified in the first simulation study except for the distribution of random effects. That is, random effects are distributed as

$$b_{i1}\overset{i.i.d}{∼}0.6N\left(-0.8,0.5\right)+0.4N\left(1.2,0.5\right)\;\;\mathrm{and}\;\;b_{i2}\overset{i.i.d}{∼}0.6N\left(0.8,0.5\right)+0.4N\left(-1.2,0.5\right),$$

where random effects have bimodal features with 0.

**Figure 1:**
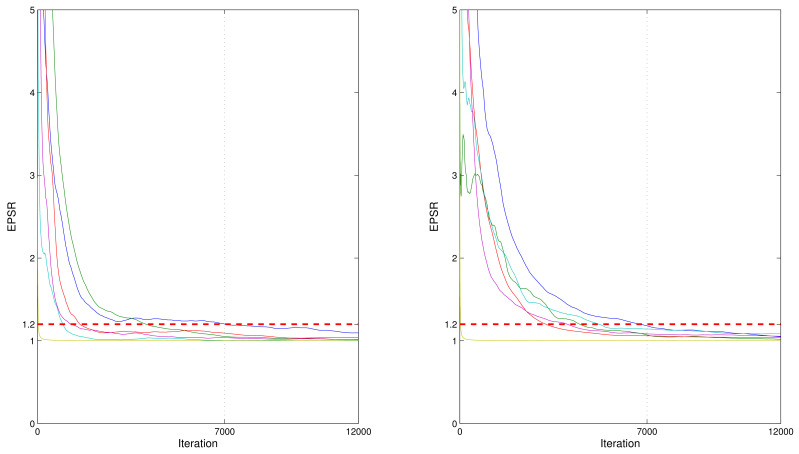
EPSR values of all parameters against iteration numbers for a randomly selected replication in the first simulation (**left** panel) and second simulation (**right** panel).

Fore each dataset generated from the abovementioned two simulation studies, the hybrid algorithm combining the block Gibbs sampler and the Metropolis–Hastings algorithm in conjunction with the BLasso method and the stick-break prior of CDPMM was used to produce Bayesian estimates of parameters and random effects as well as simultaneously select the important covariates. To investigate the convergence for these Bayesian algorithms, we computed the estimated potential scale reduction (EPSR, proposed by Gelman et al. [27]) of parameters via three parallel sequences of observations based on three different initial values. It can be seen from Figure 1 that the EPSR values were less than 1.2 after about 7000 iterations in both simulations for all the test runs. Therefore, L=5000 observations collected after 7000 iterations were used to compute the simulation results for all replications. Results obtained under simulations 1 and 2 are reported in Table 1, where ‘Bias’ is the difference between the true value and the mean of the estimates based on 500 replications; ‘RMS’ is the root mean square of differences between the true values and their corresponding estimates based on 500 replications. Compared with the Lasso from the frequentist view, the BLasso would not shrink the non-significant elements of β exactly toward 0 since the sampling-based method is involved. Thus, as suggested by Tang et al. [20], the criterion for variable selection is that a coefficient is viewed as 0 if its 95% confidence interval includes zeros. In Table 1, ‘F0’ denotes the proportion that the number of 95% confidence interval for regression parameter including zero in 500 replications is divided by 500. The larger the values of F0 corresponding to non-significant regression parameters, and the smaller the values of F0 corresponding to significant parameters, the better the performance of the posited model.

**Figure 2:**
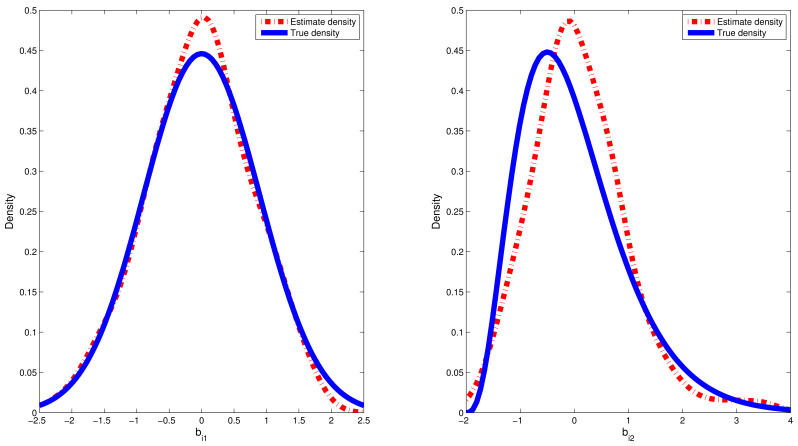
Estimated densities versus true densities for random effects $b_{i1}$ and $b_{i2}$ in the first simulation.

**Figure 3:**
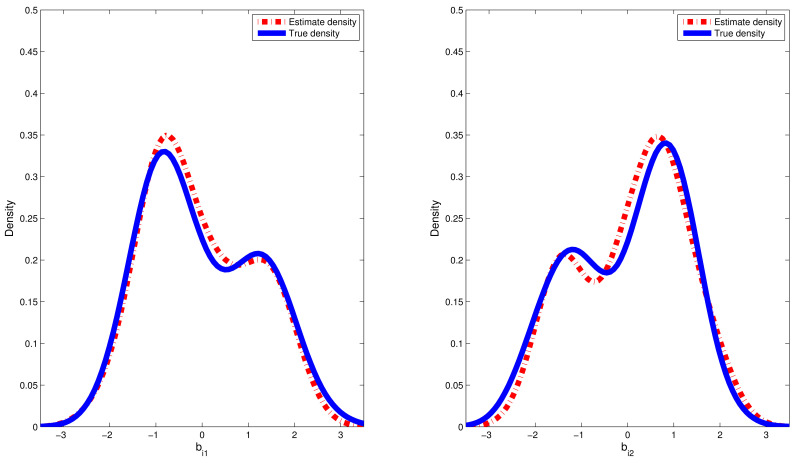
Estimated densities versus true densities for random effects $b_{i1}$ and $b_{i2}$ in the second simulation.

Examination of Table 1 indicated that (i) the Bayesian estimates of the unknown parameters β and $\sigma^{2}$ were reasonably accurate under the two abovementioned simulation studies since their absolute biases were less than 0.10 and their RMS values were less than 0.16; (ii) BLasso could correctly identify the zero and nonzero coefficients in most cases because the F0 values corresponding to important covariates were less than 10%, whilst the F0 values corresponding to unimportant covariates were near to 90%. On the other hand, to investigate the effectiveness of using the CDPMM prior for the random effects, we introduced the following RMSE (root of mean squared error) criterion in term of random effects,

$$\mathrm{RMSE}\left({\hat{b}}_{i}\right)=\sqrt{\frac{1}{2L}\sum_{m=1}^{2}\sum_{l=1}^{L}{\left(p_{m}\left(h_{l}\right)-{\hat{p}}_{m}\left(h_{l}\right)\right)}^{2}},$$

where $p_{m}\left(\cdot \right)$ and ${\hat{p}}_{m}\left(\cdot \right)$ denote, respectively, the true density function for random effect $b_{im}$ and kernel density estimation for the estimated values of random effect $\left\{{\hat{b}}_{im}:i=1,\ldots ,n\right\}$; $h_{l}$ is chosen to be the $\frac{l}{L}$ th quantile of the dataset $\left\{{\hat{b}}_{im}:i=1,\ldots ,n\right\}$. The sample quantiles for the estimated values of RMSE are reported in Table 2. Furthermore, we chose a typical replication whose RMSE value is equal to the median in the 500 replications. Therefore, on the basis of the selected replication, the estimated densities of $b_{i1}$ and $b_{i2}$ based on the CDPMM prior against their corresponding true densities are plotted in Figure 2 and Figure 3, which indicated that the finite mixture of normal distributions can flexibly capture the symmetric, skewed and bimodal shapes of random effect $b_{i}$. From Table 2, based on the results of 500 replication in both simulations, the estimated means and standard deviation (SD) of random effects $b_{i1}$ and $b_{i2}$ is approximate to their corresponding true values, the 25%, 50% and 75% quantiles of $\mathrm{RMSE}({\hat{b}}_{i})$ values are small and close enough, which indicated that it is robust to apply CDPMM method to estimate random effects. All these findings indicated that (i) our proposed Bayesian procedure could capture the true information of $b_{i}$ well, regardless of their true distributions and forms, and (ii) BLasso could identify the true model with a high probability.

To compare the performance of the CDPMM prior for the random effects with the discrete DP given by Ishwaran and Zarepour [26] and Yang et al. [25], we conducted the following third simulation study. In this simulation study, 500 simulated datasets were generated by using the same setup as specified in the first simulation study, and fitted by the model with the discrete DP for the random effects. In the fourth simulation, we reanalyzed the aforementioned 500 datasets generated in the second simulation by using a parametric Bayesian approach with the random effects distribution specified by a multivariate normal distribution. The aim of this simulation was to compare the semiparametric approach based on the CDPMM prior with the parametric approach based on the Gaussian prior from the Bayesian perspective. Results obtained under the third and fourth simulations are reported in Table 3. Our programs were written in Matlab. It roughly took 119.3 s and 186.9 s in an Intel(R) Xeon(R) Silver 4216 CPU @ 2.10GHz (Intel, Santa Clara, CA, USA) serve to run 12,000 iterations for our proposed CDPMM and discrete DP, respectively; this indicates that the CDPMM method is much more efficient than the discrete DP in our considered simulations. It can be seen from Table 2 and Table 3 that (i) our proposed CDPMM and discrete DP methods have the same performance in term of the ’Bias’ and ’RMS’ values, but the F0 values corresponding to the non-significant parameters under the proposed CDPMM prior are higher than those under the discrete DP prior; (ii) the RMS values and the correct rates of variable selection based on the F0 values under the semiparametric CDPMM prior are better than those under the parametric Gaussian prior.

### 4.2. Real Example

In this section, the application of our proposed approach to the skewed longitudinal proportional data is illustrated by the analysis of a prospective ophthalmology study [28] from the Bayesian perspective. The prospective ophthalmology data used in the study were obtained from the Supplementary Materials of a paper by Song and Tan [29] and were available from https://biometrics.biometricsociety.org/home/archive/supplementary-materials, accessed on 5 september 2022. This prospective ophthalmology study described that the eyes of 31 patients before surgery were injected by three gas concentration levels of $C_{3}F_{8}$, and all patients after surgery were followed up three–eight times over a three-month period. The outcome variable was the percentage of remaining gas volume relative to the initial volume of gas injected. These longitudinal proportional data from a prospective ophthalmology study were analyzed by Qiu et al. [1], Song and Tan [29] and Song et al. [30], respectively. However, these authors did not consider to conduct variable selection in the analysis of this dataset. Our scientific interest in this study is to investigate the effect of three initial gas concentration levels of $C_{3}F_{8}$ and time on the percentage of remaining gas volume, while accounting for selecting the important covariates based on the BLasso method. Let the response $y_{ij}$ denote the percentage of gas left in the eye for patient *i* at the *j*th follow-up day, $t_{ij}$, and $y_{ij}|b_{i}∼S^{-}\left(\mu_{ij},\sigma^{2}\right)$. Thus, the conditional mean in our proposed semiparametric mixed-effects model is given by

$$logit\left(\mu_{ij}\right)=\beta_{0}+\log\left(t_{ij}\right)\beta_{1}+\log^{2}\left(t_{ij}\right)\beta_{2}+\beta_{2}w_{ij}+b_{i1}+\log\left(t_{ij}\right)b_{i2}$$

where $t_{ij}$ is the time covariate for days after the gas injection; $w_{ij}$ is the covariate of gas concentration levels equal to −1, 0 and 1, corresponding to the concentration levels of 15%, 20% and 25%, respectively; and random effects $b_{i1}$ and $b_{i2}$ are specified by CDPMM in (4), which characterize the effect fluctuations of interception and logarithmic time among patients.

**Figure 4:**
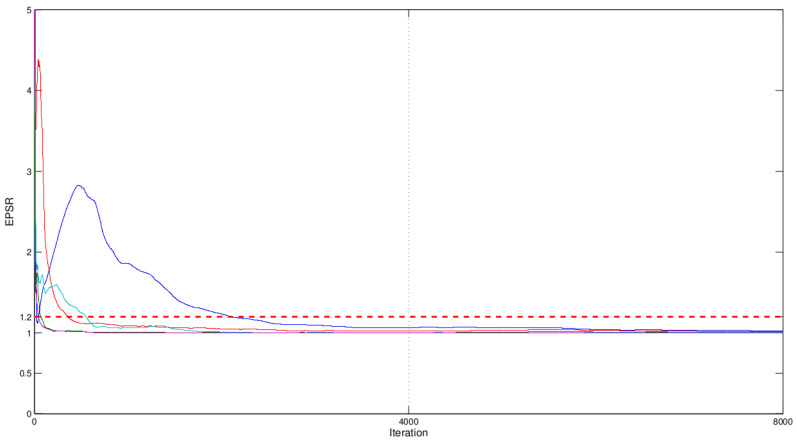
EPSR values of all parameters against iteration numbers in the ophthalmology study.

**Figure 5:**
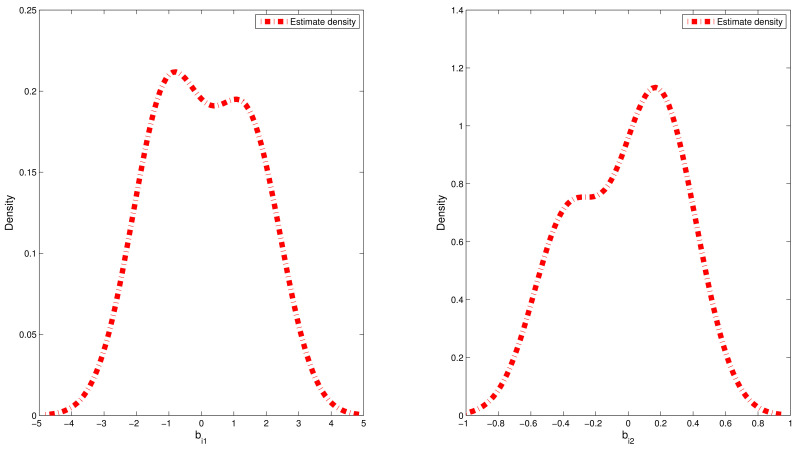
Estimated densities for random effects $b_{i1}$ and $b_{i2}$ in the ophthalmology study.

The abovementioned MCMC algorithm was used to produce the joint Bayesian estimates of parameters and random effects in this real example. In the implementation of MCMC process, the hyperparameter values were taken to be the same as those given in simulation. Similarly, we used the EPSR method given in simulation to investigate the convergence for these algorithms. The EPSR values of all parameters against the iteration numbers was plotted in Figure 4, which indicated that the MCMC algorithm converged after 4000 iterations since their EPSR values were less than 1.2 after about 4000 iterations. Hence, L=4000 observations collected after 4000 iterations were used to calculate Bayesian estimates of parameters and random effects. Results are reported in Table 4 and Figure 5, which indicated that (i) the estimated densities of random effects $b_{i1}$ and $b_{i2}$ were bimodal and skewed, which indicated that traditional normality assumption for random effects is inappropriate in this real example; (ii) the square of logarithmic time ($\log^{2}\left(t_{ij}\right)$) was detected to be an important covariate with a significantly negative effect on the percentage of gas left in the eye, since its corresponding 95% confidence interval did not include zero. The gas concentration levels ($w_{ij}$) and the logarithmic time ($\log^{2}\left(t_{ij}\right)$) were insignificant at significance level 0.05 because their 95% confidence interval included zero.

## 5. Conclusions

In this paper, we introduced a new semiparametric simplex mixed-effects models with the random effects following the centered Dirichlet process mixture model (CDPMM). The advantages of the proposed model based on CDPMM are the following: (i) it can capture the features of skewed and bimodal longitudinal proportional data; (ii) it can characterize absolutely continuous distributions for random effects. The novelty of our approach is that we adopted the BLasso procedure to simultaneously estimate parameters of interest, provide credible intervals (CIs) for parameters and conduct both shrinkage and variable selection for our considered models. A hybrid algorithm combining the Gibbs sampler and the MH algorithm was used to simultaneously obtain Bayesian estimates of unknown parameters, random effects and their standard errors and credible intervals. Empirical results show that (i) the proposed semiparametric Bayesian method provides quite accurate estimates of parameters (see Table 1); (ii) the average frequencies of correctly identifying unimportant predictors were near to 90%; (iii) CDPMM can effectively capture the potential features of normal, gamma and mixture normal distributions (see Table 2 and Figure 2, Figure 3 and Figure 5).

## Figures and Tables

**Table 1:** Bayesian estimates of parameters in the first and second simulation studies.

	Simulation 1			Simulation 2			
**Par.**	**True**	**Bias**	**RMS**	**F0 (%)**	**Bias**	**RMS**	**F0 (%)**
$\beta_{0}$	−0.45	0.054	0.117	1.60	0.031	0.143	5.40
$\beta_{1}$	0.00	0.007	0.109	89.20	0.002	0.105	88.20
$\beta_{2}$	0.45	0.017	0.116	1.40	0.018	0.132	3.00
$\beta_{3}$	−0.00	−0.001	0.141	94.60	−0.003	0.153	93.40
$\beta_{4}$	0.45	−0.074	0.136	3.80	−0.051	0.146	8.20
$\sigma^{2}$	1.00	0.006	0.070	—–	0.009	0.073	—–

Note: Bias denotes the difference between the true value and the mean of the estimates based on 500 replications; RMS denotes the root mean square of differences between the true values and their corresponding estimates based on 500 replications; F0 denotes the proportion of the 95% confidence interval for regression parameter including zero in 500 replications.

**Table 2:** Estimated means, standard deviations and RMSE quantile of random effects in the first and second simulation studies.

Random Effects	Simulation 1				Simulation 2			
	**Mean**	**Est Mean**	**SD**	**Est SD**	**Mean**	**Est Mean**	**SD**	**Est SD**
$b_{i1}$	0.000	0.040	0.894	0.831	0.000	−0.030	1.100	1.032
$b_{i2}$	0.000	0.063	1.000	0.903	0.000	0.031	1.100	0.995
	**Quantile of Simulation 1**				**Quantile of Simulation 2**			
	5%	25%	75%		5%	25%	75%	
RMSE($b_{i}$)	0.031	0.039	0.050		0.079	0.084	0.095	

Note: ’Mean’ denotes true empirical mean of the distribution; ’Est mean’ denotes mean of the posterior samples; ’SD’ denotes true empirical standard deviation of the distribution; ’Est SD’ denotes standard deviation of the posterior samples.

**Table 3:** Bayesian estimates of parameters in the third and fourth simulation studies.

		Simulation 3			Simulation 4		
**Par.**	**True**	**Bias**	**RMS**	**F0 (%)**	**Bias**	**RMS**	**F0 (%)**
$\beta_{0}$	−0.45	0.005	0.101	1.20	0.064	0.151	8.40
$\beta_{1}$	0.00	0.000	0.104	86.60	−0.009	0.130	84.40
$\beta_{2}$	0.45	−0.001	0.123	1.40	0.033	0.148	3.80
$\beta_{3}$	−0.00	0.005	0.166	89.20	0.007	0.185	90.00
$\beta_{4}$	0.45	−0.003	0.119	2.00	−0.068	0.143	11.40
$\sigma^{2}$	1.00	0.093	0.134	—–	0.006	0.078	—–

Note: Bias denotes the difference between the true value and the mean of the estimates based on 500 replications; RMS denotes the root mean square of differences between the true values and their corresponding estimates based on 500 replications; F0 denotes the proportion of the 95% confidence interval for regression parameter including zero in 500 replications.

**Table 4:** Bayesian estimates (BEs) and standard deviations (SDs) and 95% credible intervals (CIs) for parameters in the ophthalmology study.

Par.	BE	SD	CI
$\beta_{0}$	2.579	0.257	(2.200, 3.031)
$\beta_{1}$	0.093	0.172	(−0.232, 0.427)
$\beta_{2}$	−0.322	0.045	(−0.412, −0.241)
$\beta_{3}$	0.368	0.281	(−0.131, 0.697)
$\sigma^{2}$	9.664	1.317	(7.406, 12.516)

## Data Availability

The research data are available on the website: https://biometrics.biometricsociety.org/home/archive/supplementary-materials, accessed on 5 september 2022.
